# Carbon quantum dots: An environmentally friendly and valued approach to sludge disposal

**DOI:** 10.3389/fchem.2022.858323

**Published:** 2022-08-11

**Authors:** Bruno L. Rossi, Cláudia M. B. Andrade, Eralci M. Therézio, Romildo J. Ramos, Leonardo G. Vasconcelos, Ailton J. Terezo, Adriano B. De Siqueira

**Affiliations:** ^1^ Genmat/Rede MT-NanoAgro- Departamento de Química/ICET, Universidade Federal de Mato Grosso, Cuiabá, MT, Brazil; ^2^ Instituto de Física, Universidade Federal de Mato Grosso, Cuiabá, MT, Brazil

**Keywords:** nanomaterial, sustainability, thermal analysis, luminescence, sewage sludge

## Abstract

Sewage sludge, produced daily and inherent to urban development, presents problems of disposal that are still challenging today. Its disposal still offers palliative solutions, where the final destination is generally in landfills or, restrictively, to use in agriculture. The synthesis of carbon quantum dots (CQDs) from sewage sludge is a better alternative to use the stock of organic material present in the sludge. The present work aims to produce Carbon quantum dots (CQDs) using principles of green chemistry and to use an alternative raw material intrinsic stock of carbon present in sewage sludge, making its final disposal more sustainable. The material obtained has a core structure mainly composed of sp^2^ carbon and nitrogen. The surface functional groups containing sulfur, nitrogen, and oxygen of CQDs were investigated using FTIR and TG/DSC coupled FTIR techniques. The CQDs showed a luminescence decay time equivalent to fluorescent compounds and with satisfying quantum yield since no passive/oxidizing agent or material purification process was used. The photoluminescence spectroscopy analysis showed that the CDQs excitation λ_max_ was at 360 nm and caused a λ_max_ emission at 437 nm (CQDs^a^) and 430 nm (CQDs^b^). The CQDs obtained showed sizes of 9.69 ± 2.64 nm (CQDs^a^) and 10.92 ± 2.69 nm (CQDs^b^). *In vitro* experiments demonstrated the uptake of CQDs by the endothelial cell line EAhy 926 and their nontoxicity. However, the production of CQDs can be used for the sustainable disposal of sewage sludge.

## Introduction

Currently, the disposal of sludge and even its treatment is as complex as the treatment of liquid sewage, representing more than 50% of the total cost of treatment in some sewage treatment plants (Callegari and Capodaglio, 2018). In Brazil, adopting an average rate of generation of sewage sludge equivalent to 30 g per inhabitant per day, it is estimated that approximately 3,090 tons/day of sludge was generated at sewage treatment plants (STP) operating in 2017 (De and SNS., 2017). The lack of adequate sludge treatment may compromise the environmental benefits conferred by sewage treatment. The accumulation of sewage sludge in landfills requires more and more territorial space, in addition to contaminating the soil, water table and the air of people who live around the dump site. The most common options for the final disposal of sewage sludge have generally been disposal in landfills, agricultural use, and incineration (Peccia and Westerhoff, 2015; Callegari and Capodaglio, 2018; Collivignarelli et al., 2019).

Chemical analysis of sewage sludge shows that its organic fraction is a mixture, where the most significant fraction is protein, lignin, and lipids, and to a lesser extent carbohydrate, cellulose, and hemicellulose (Callegari and Capodaglio, 2018). Thus, although the composition of the sludge varies according to the characteristics of the treated sewage, a substantial fraction has in its composition a significant portion of organic material, which arouses interest for the production of carbon quantum dots (CQDs) (Peccia and Westerhoff, 2015; Callegari and Capodaglio, 2018; Collivignarelli et al., 2019; Monje et al., 2021).

Studies have also shown that CQDs have both sp^3^ and sp^2^ carbon moieties in their structure, and they can be used as fluorescent cell markers and for intracellular drug delivery (Krysmann et al., 2012; Sahu et al., 2012; Yang et al., 2014a; Liu et al., 2018). CQDs have gained importance globally due to their photoluminescence, photostability, biocompatibility, hydrophobicity, low-cost synthesis methods, and low toxicity (Baker and Baker, 2010; Yang et al., 2013; Guo et al., 2015; Yuan et al., 2016; Zhang and Yu, 2016). CQDs are promising candidates for biosensors, bioimaging, chemical analysis, cancer therapy, photocatalysis, gene transfer, optoelectronic devices and solar cells (Wang and Hu, 2014a; Guo et al., 2015; Yuan et al., 2016; Tuerhong et al., 2017). The raw materials used for the production of CQDs may be pure compounds such as citric acid and folic acid or more complex organic systems such as honey (Yang et al., 2014b), banana juice (De and Karak, 2013), lime (Barati et al., 2015), frying oil (Hu and Yang, 2014), gelatin (Fang et al., 2012), silk cocoons (Li et al., 2013), grass (Liu et al., 2012), flour (Qin et al., 2013), and saffron (Ensafi et al., 2017a). Different sizes of CQDs can be obtained for various applications. The larger their size is the more significant the shift to the photoluminescent spectrum in the red region (Wang and Hu, 2014b; Tuerhong et al., 2017; Zhao et al., 2019).

The Sewage sludge from the Liede subdistrict in Guangzhou, China, was used to prepare CQDs. The dry sludge was treated with hydrogen peroxide and autoclaved at 180°C for 12 h, obtaining a photoluminescent solution with 10.3% quantum yield (QY) (Pires et al., 2015). Even though it is more difficult to obtain CQDs with high QYs from biomass and residues, the commitment to develop synthesis routes using these materials is desirable because, in addition to being abundant, it will lead to a reduction in the amount of waste disposed of in landfills/dumps (Baker and Baker, 2010; Pedroza et al., 2010).

The solvothermal method is most commonly used process in the preparation of CQDs, it utilizes the solubility of the precursors in a solvent at high temperatures and pressures. Therefore, investigations into the effects of microwave treatment duration on precursors of pure substances have shown that increasing time increases the average size of CQDs (Meiling et al., 2016a). Other parameters of synthesis that have been extensively studied are variations in the precursor concentration, time, temperature, and in some cases, the amount of oxidizing agent. In the synthesis of CQDs using Chinese sewage sludge, these parameters were investigated. They found that a higher temperature and a longer reaction time led to an increase in QY. However, the concentration of sewage sludge and the oxidizing agent have an optimal concentration that negatively impacts QY when exceeded (Pires et al., 2015). The pyrolysis route for the production of CQDs using one or more types of biomass can be obtained through the thermogravimetric profile of the raw materials (Monje et al., 2021).

Considering that CQDs are an emerging star nanomaterial, they have exhibited more advanced properties and are currently being developed for various applications owing to their unique properties, such as multicolor emissions, excellent photostability, and biocompatibility, and easy surface functionalization. The advantages of CQDs allow them to be widely used in biosensors, photocatalysis, biomedicine, and more recently, agriculture (Agnol et al., 2021; Li et al., 2021; Goswami and Mathur, 2022). The work elaborated here seeks an innovative approach to using stabilized sludge as a carbon precursor material to produce CQDs, without the need for oxidizing agents, reducing agents, passivation, or other purification processes. The prospect for further applications being able to produce CQDs on a large scale using domestic sewage sludge will guarantee sustainable disposal with immeasurable socioeconomic-environmental value to society. Furthermore, this work and its sequence can be an important tool to reach the 2030 agenda for sustainable development and sustainable development goals (SDGs).

## Experimental

### Synthesis

The sewage sludge was collected in an upflow anaerobic sludge blanket (UASB) reactor that is part of the sewage treatment plant of a hotel in Cuiabá-MT, Brazil.

The sewage sludge was previously treated using a 5804 R Eppendorf centrifuge, set at 2,000 rpm for 20 min. The supernatant was discarded and the solid obtained was called wet sludge, which was stored in the freezer at −18°C for later use. The wet sludge was dried in an oven with air circulation at 80°C for further characterization.

The wet sludge quantities of wet sludge were used with different masses according to the concentration to be indicated by the factorial design and mixture with 40 ml of distilled water. These mixtures were subjected to two solvothermal treatments.

1) Microwave treatment was performed using a sample system digester, Speedwave 4 model (Berghof), using twelve 60 ml Teflon flasks for synthesis. The irradiation power was set to 800 W using 80% power, and the pressure was set to 40 bar.
2) A steel autoclave with a 50 ml Teflon chamber was used in the hydrothermal treatment, and the ensemble was heated in a muffle furnace. The temperature and time were established as indicated in the factorial design.

The products obtained by both treatments were cooled to room temperature and then centrifuged using a 5804 R Eppendorf centrifuge at 11,000 rpm for 20 min. The supernatant was filtered using a 42 grid paper filter (Whatman) with 2.5 µm pores and further characterization. The luminescence of the materials was evaluated with a lamp emitting at 360 nm.

### Factorial design

The factorial design was performed using preliminary tests. First, to analyze the individual effects, the interactions between the independent variables were analyzed. Then, the syntheses of the CQDs were performed using a complete 3^3^ factorial design to verify the influence of the variables temperature, concentration, and exposure time to microwave radiation (CQDs^a^). Synthesis temperature levels were 150°C, 180°C, and 210°C. The concentration levels of diluted wet sludge in distilled water were 6.25 g L^−1^, 12.50 g L^−1^, and 18.75 g L^−1^. The microwave digester exposure times were 8, 15, and 30 min. Finally, the influence of the variables was evaluated using quantum yield (QY) as a response in the planning study.

An experiment with seven trials was performed to verify the synthesis of CQDs obtained by hydrothermal treatment in a muffle heated autoclave (CQDs^b^). The synthesis was performed by varying the exposure time (3, 4, 6, 8, 10, 12, and 18 h) and keeping the concentration and temperature fixed at 6.25 g L^−1^ and 210°C.

### Characterization

The absorption spectra of the samples were measured using a Varian Cary model 50 Bio Xenon pulsed source UV-Vis spectrophotometer. Measurements were taken at room temperature and the scan comprised excitation wavelengths from 200 to 800 nm. The samples were arranged in quartz cuvettes with a 10 mm optical path for analysis.

The emission spectra of the samples were measured using an ISS PC1 photon-counting spectrofluorometer, operating with a 300-W xenon lamp powered by a stabilized current of 15 A. The excitation and emission monochromators for sample measurements used a spectral slit width of 1.0 mm. The excitation wavelength was increased by 20 nm, spanning the range 280–500 nm, and the emission was recorded from 300 to 700 nm. The samples for emission analysis were arranged in quartz cuvettes with a 10 mm optical path.

The determination of the QY of the CQD diluted samples was performed using the values of the integrated photoluminescence intensity and the absorbance of the samples and related to standard quinine sulfate, as indicated in the literature (Wang and Hu, 2014b). The quinine dihydrate sulfate used was from Fluka, with a molecular weight equal to 782.96 g mol^−1^; this material was previously prepared in 0.1 mol L^−1^ H_2_SO_4_. An acid solution of quinine sulfate was used with a molar concentration equal to 1.10^−5^ mol L^−1^.

The thermal analysis was performed on a Shimadzu DTG-60H thermal analyzer. The experiment was performed in a dry air atmosphere with a flow rate equal to 100 ml min^−1^, and a heating ratio equal to 20°C min^−1^, using crucibles of platinum and alumina, with samples weighing approximately 10 mg. The evolved gas analysis was performed on a Mettler TG-DSC 1 thermogravimetric analyzer coupled to an iS10 Nicolet FT-IR spectrometer. The experiment was performed in a N_2_ and dry air atmosphere at a flow rate equal to 60 ml min^−1^, and a heating ratio equal to 20°C min^−1^, using alumina crucibles, with samples weighing approximately 20 mg. The transfer line consisted of a 120 cm long, 2 mm internal diameter glass-lined stainless-steel tube heated to a constant temperature of 225°C. FTIR measurements were performed with a DTGS detector in a specially developed gas cell, heated to a constant temperature of 250°C. The interferometer and gas cell compartments were purged with N_2_ and air. FTIR spectra were recorded in the 4,000–400 cm^−1^ range, comprising 16 spectrum scans at a resolution of 4 cm^−1^.

The infrared absorption spectra of the lyophilized CQDs were obtained using a Nicolet Fourier transform spectrophotometer, model iS10, with a resolution of 4 cm^−1^, in the region between 4,000–600 cm^−1^, using the attenuated total reflectance (ATR) technique. For this analysis, the sludge samples were oven-dried at 110°C for 24 h.

Images of materials synthesized in a microwave and autoclave were obtained using a Philips transmission electron microscope model CM 200 operating at 120 kV with a LaB6 filament. Samples were prepared on copper grids (TED PELLA, ultrathin C type A, 400 mesh). The sample was dispersed in water and kept in an ultrasound bath for 10 min for redispersion. One microliter was dropped onto the copper grid, which was dried at room temperature for analysis. The camera constant used was 1,210 pxl/A.

### Cellular experiments

To test the applicability of CQDs^a^, we evaluated cellular uptake and toxicity *in vitro*. The experiments were performed on the endothelial cell line EAhy 926, kindly provided by Dr. Carmen Lúcia Bassi Branco. Cells were seeded on glass coverslips and cultured with high glucose Dulbecco’s Modified Eagle’s Medium (DMEM) supplemented with 10% fetal bovine serum, 4 mmol L^−1^ glutamine, 1 mmol L^−1^ sodium pyruvate, and 1,500 mg L^−1^ sodium bicarbonate. Afterward, the cultures were incubated with CQDs (0.4 mg ml^−1^) for 1 h. After the residual particles were removed, the nuclei were stained with 4′,6-diamidino-2-phenylindole (DAPI). Cell viability was assessed by staining with trypan blue. Finally, images of cultures treated or not with CQDs were acquired using an AxioScope A1 microscope (Carl Zeiss, GR) with Axiovision Software (Carl Zeiss, GR).

## Results and discussion

A qualitative test was carried out with the CQDs produced by the centrifuged sewage sludge. The analysis was performed due to a high amount of organic matter (OM). The solutions obtained were dark brown (Supplementary Figure S1), which is due to the high concentration of the precursor used. Therefore, the concentrated solution showed slight luminescence when exposed to 312 nm UV light. In addition, the high concentration of organic carbon in the solution likely promoted a more significant state of aggregation. The organic molecules present in the sewage sludge were converted into CQDs through carbonization. This approach is called bottom-up.

The solutions obtained from the synthesis of CQDs by microwave (CQDs^a^) and autoclave (CQDs^b^) treatment of wet sludge were pale-yellow when observed under white light and bright blue when subjected to 312 nm UV light.

**FIGURE 1:**
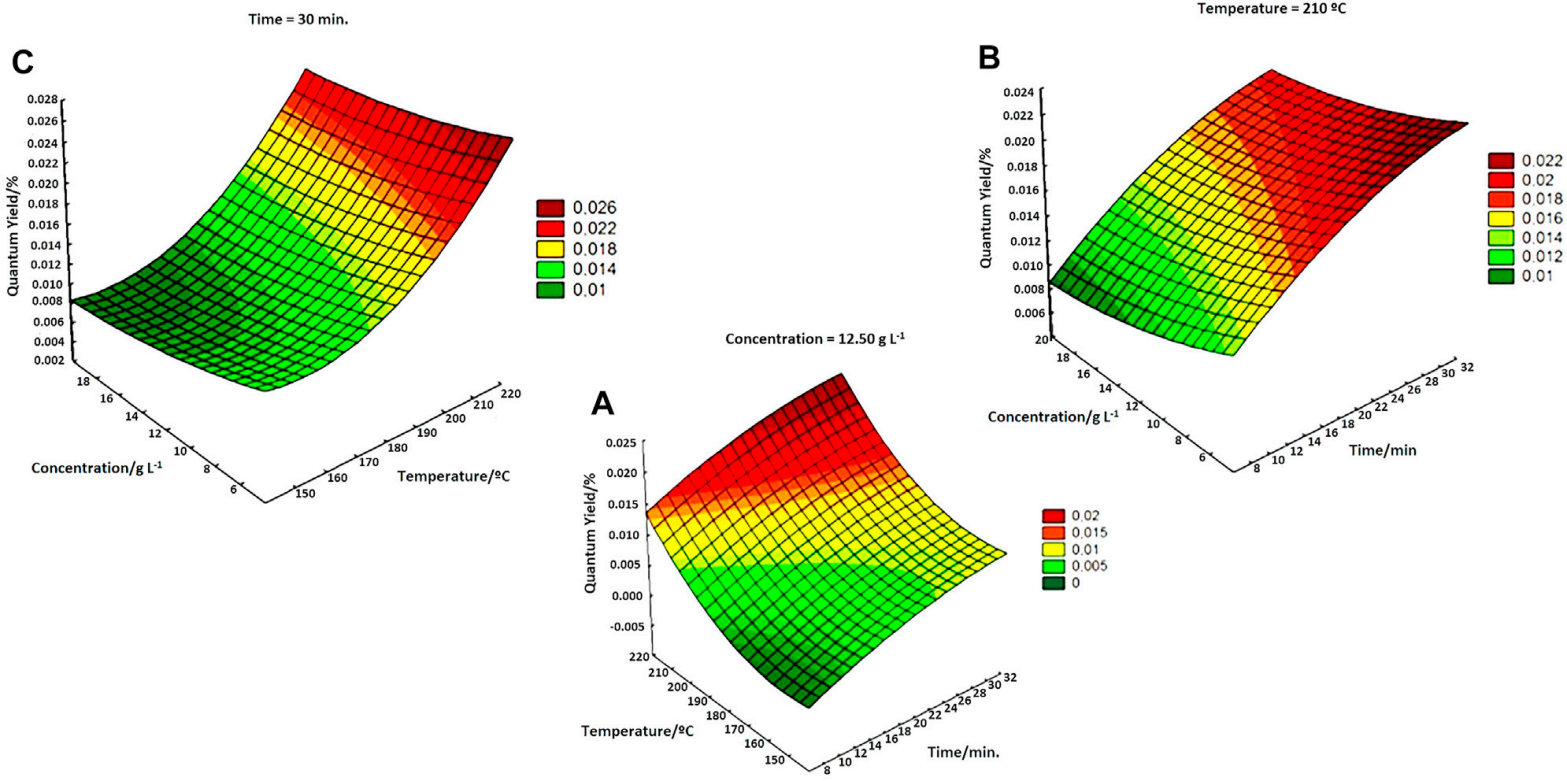
**(A)** Variation in QY at a fixed concentration of 12.50 g L^−1^ as a function of temperature and synthesis time. **(B)** Variation in QY with a fixed temperature at 210°C and varying concentrations and synthesis times. **(C)** Variation in QY with a fixed time of 30 min varying the concentration and synthesis temperature.

The significant effects of the synthesized materials were performed by quantitative analysis of the QY of the produced CQDs. The factorial experimental design was necessary, as the sludge has a complex and heterogeneous composition, which varies with the composition of the sewage that feeds the STP and can result in undetermined errors. Figure 1 shows, the variation in QY when maintaining the variables concentration, temperature, and exposure time of fixed synthesis.

The significant variables observed were temperature (T) and time (t), indicating a positive effect. Therefore, higher T and t improved the quantum yield of CQDs^a^, according to the literature (Himaja et al., 2014; Ensafi et al., 2017b). From the results obtained by the factorial design, it was possible to fix the temperature and wet sludge concentration values to vary the synthesis time by hydrothermal treatment in the autoclave and thus compare the QY value with the values obtained in microwaves.

Autoclave treatment showed that an increase in treatment time improved QY, but when the heat exposure time was greater than 10 h, a decline in this value was noted (Supplementary Figure S2). According to the QY results for CQDs^a^, high temperatures and prolonged MW radiation exposure times positively reflected the QY, and the typical concentration had no significant influence on the QY. Therefore, the microwave exposure time increased to values higher than those established in the experimental planning to verify the quantum yield (Supplementary Figure S3).

**FIGURE 2:**
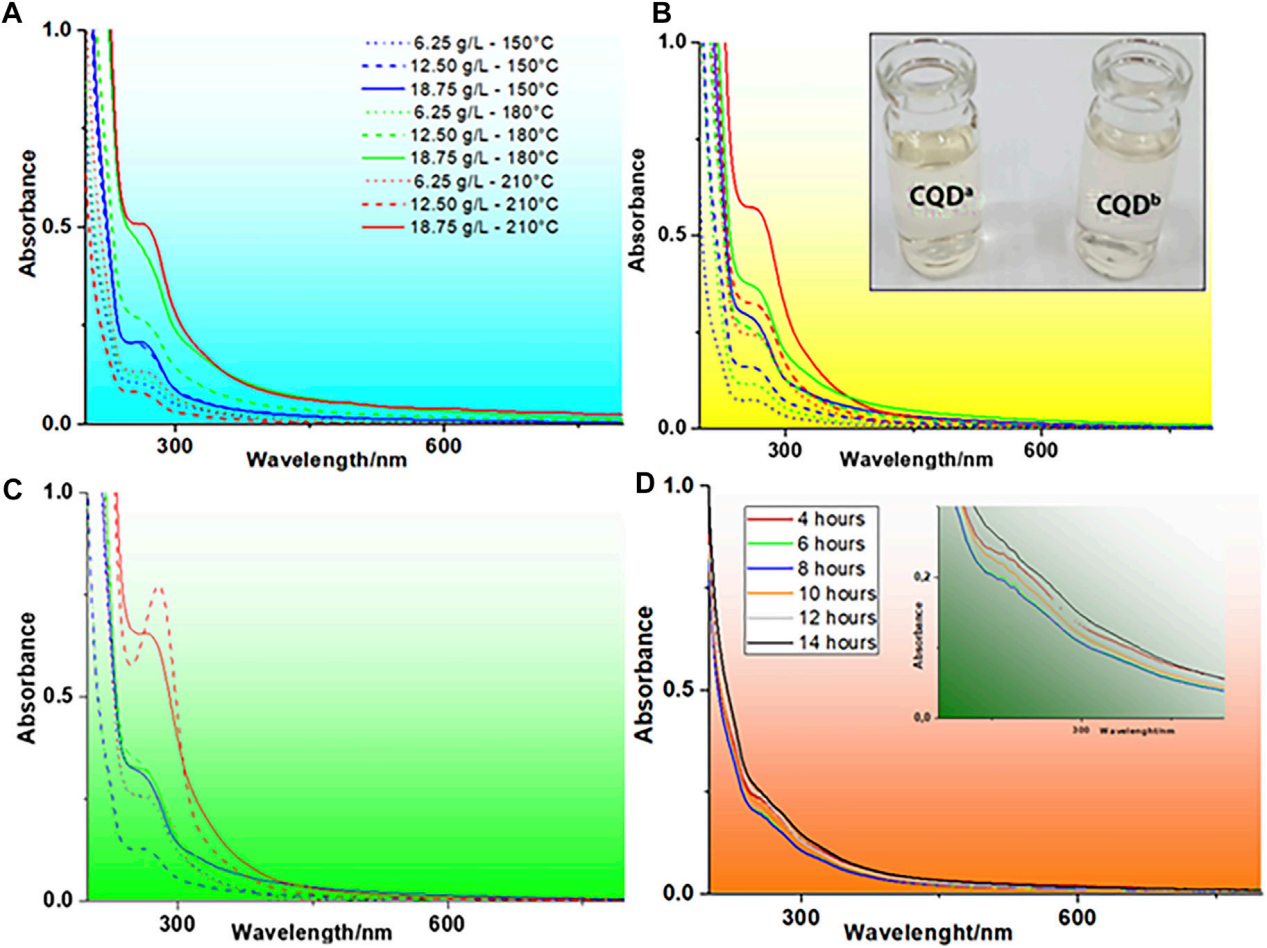
UV-Vis absorption spectra of CQDs^a^ at different concentrations and temperatures for microwave synthesis times of **(A)** 8 min, **(B)** 15 min, and **(C)** 30 min **(D)** UV-Vis absorption spectrum of CQDs^b^ at different autoclave synthesis times (using constant temperature and concentration), with absorbance magnification in the range of 220–380 nm.

It is important to note that the optimal parameters indicated the formation of a carbon core with surface formation of functional groups more efficiently for the luminescence of CQDs. The best QY (2.67%) obtained by a hydrothermal treatment was comparable to the best value obtained by microwave synthesis (2.59%), but the use of microwave radiation was more efficient, reducing the synthesis time by up to 20-fold (autoclave = 10 h; microwave = 0.5 h). The spectroscopic analyses of absorption in the UV-Vis region performed on samples treated with the microwave system can be seen in Figure 2.

### UV-Vis spectra

Figure 2 shows an absorption band at approximately 270 nm, with a tail extending into the visible region to approximately 580 nm. This absorption corresponds to the π-π* electronic transition in conjugate systems, indicating C-N and C-O functional groups and sp^2^ carbon core. Typically, in CQDs obtained in aqueous solution, a band of λ_max_ close to 350 nm can also be observed, which is characteristic of the n-π* electronic transition of carbonyls (Qin et al., 2013; Himaja et al., 2014); however, in the UV-Vis spectra observed in Figure 2, this band cannot be observed due to other overlapping chromophore groups and the bathochromic effect which is more pronounced for π-π*. Commonly, the absorption of the -CN group occurs in the range between 213 and 500 nm. The tail observed in the UV-Vis spectra can be explained by the transition of the C-N group and the number of nonuniform conjugate systems (Xu et al., 2011; Hsu and Chang, 2012; Himaja et al., 2014; Liu et al., 2014).

The results also show that there is a direct relationship between increased absorbance and carbon core formation. This phenomenon is probably due to the higher carbonization of sedimented sludge in the processed sample and, therefore, better synthesis yield of CQDs^a^. The results obtained for CQDs^b^, at different times of autoclave exposure, keeping the concentration and temperature constant, are presented in Figure 2D. The analysis of CQDs^b^ showed discrete absorption peaks at approximately 260 and 320 nm. The difference observed in the UV-Vis spectra of CQDs^a^ and CQDs^b^ suggests that the synthesis pathway of CQDs influenced the carbon nucleus structure.

### Photoluminescence

The investigation of the intensity of light emission of the CQDs obtained with different synthesis times when excited at 360 nm is presented in Supplementary Figure S4. The CQDs obtained by both routes showed peak emission (437 nm by microwave and 430 nm by autoclave) when excited at 360 nm. An increase in microwave synthesis time from 4 to 8 h gradually increased the emission intensity. However, at a synthesis time of 0.5 h, the produced CQDs had an intensity between the 6- and 7-h curves, with no direct relationship with the other results. In Supplementary Figure S4, it is possible to observe that microwave synthesis causes, on average, a redshift in the CQDs emission, which may imply different CQDs sizes formed or an increase in impurities (Padilha et al., 2013; Lui et al., 2019; Aoki et al., 2021). It is also noteworthy that the CQDs size does not depend on the synthesis time, as the emission is stable at maximum energy. However, as the synthesis time increases, the PL intensity decreases, probably due to the formation of traps or aggregates in the CQDs (Padilha et al., 2013; Lui et al., 2019; Aoki et al., 2021).

**FIGURE 3:**
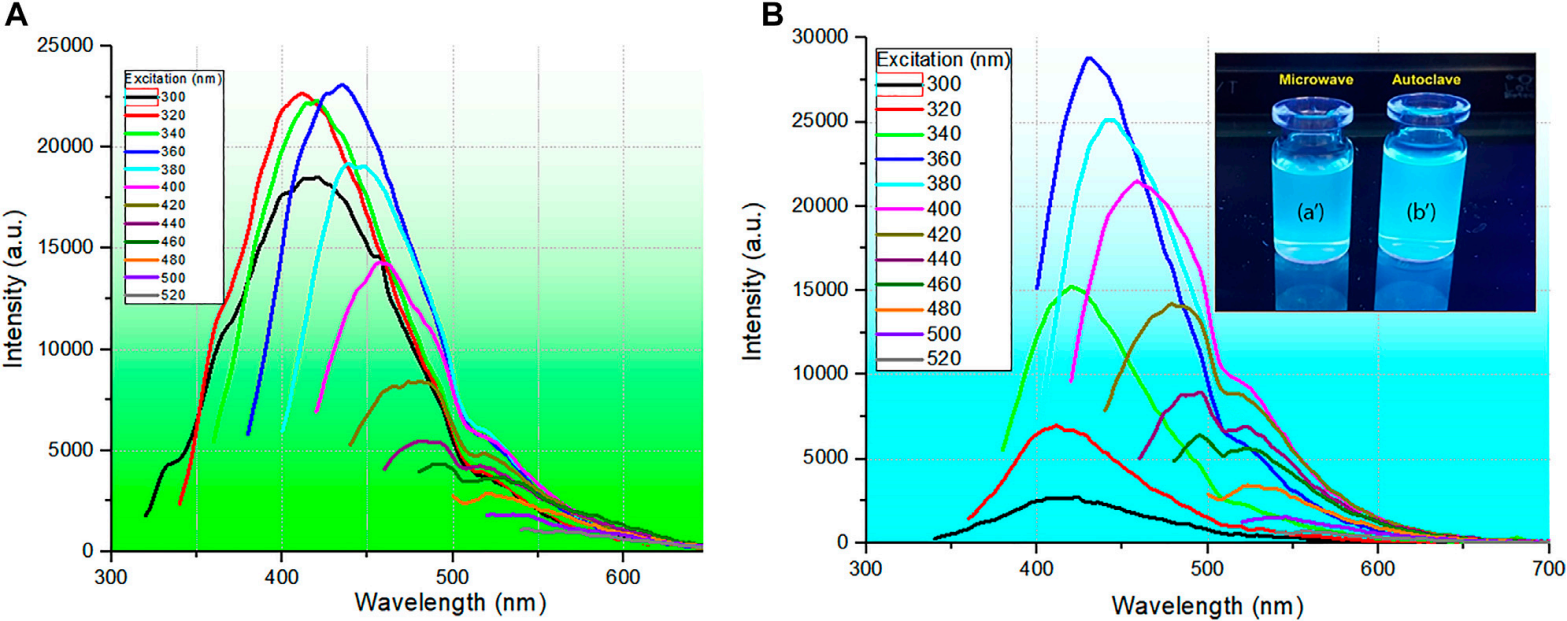
Photoluminescence spectra of CQDs synthesized **(A)** with 800 W microwave radiation at a temperature of 210°C for 30 min and a wet sludge concentration of 6.25 g L^−1^ and **(B)** in the autoclave system at a temperature of 210°C for 10 h and a wet sludge concentration of 6.25 g L^−1^. Solutions prepared by microwave (a′) and autoclave (b′) treatment under 312 nm UV light.

The results of the photoluminescence (PL) analysis of CQDs^a^ and CQDs^b^, ranging from 300 to 520 nm excitation, are presented in Figure 3.

In selective PL, when the excitation energy is changed, the dependence of the PL spectrum on the excitation energy is evident. Thus, a redshift occurs when the excitation energy is decreased (longer wavelength); in the case shown, the maximum PL changes from 410 to 510 nm. Furthermore, when excited at 320, 340, and 360 nm, the PL spectra of CDQs^a^ showed similar emission intensities, which may imply similarity in the quantum luminescence efficiency (Therézio et al., 2011; 2013) and the same energy transfer rate (Stokes shift) (Schura et al., 2019). However, in the PL spectra of CQDs^b^, when excited at these same energies (320, 340, and 360 nm), there is a substantial increase in the PL intensity as the excitation changes, certainly implying a higher luminescence quantum efficiency. This behavior is undoubtedly related to the nanoparticle sizes selected for each excitation wavelength, implying a more extensive size distribution of CQDs that will absorb the excitation energy, causing a redshift in the emission (Padilha et al., 2013). Furthermore, this behavior may be related to different types of traps produced by different synthesis routes, which may favor the homogeneity of nanoparticle sizes and, on the other hand, increase the number of traps, impairing the energy transfer of the photoexcited carriers and tending for more energy transfer in nanoparticles with larger diameters, which leads to an increase in intensity and contributes to the spectral redshift. Finally, this selective emission behavior certainly endows these materials with potential application in optical sensors and can potentially be used as bioselective markers.

### Infrared spectra

**FIGURE 4:**
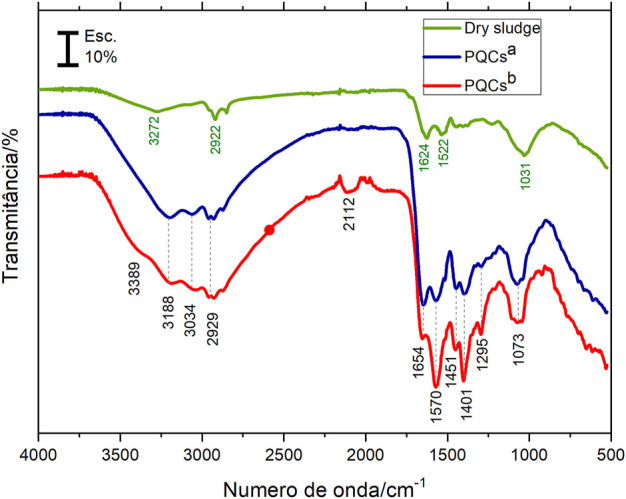
FTIR of sewage sludge used as precursor and CQDs^a^ and CQDs^b^.

The FTIR spectra of the sludge and CQDs^a^ and CQDs^b^ obtained for the optimal synthesis methods are shown in Figure 4. The bands observed with minimum transmittance values at 3,389, 3,272, and 3,188 cm^−1^ are attributed to the -N-H and -O-H groups’ vibration stretches and hydrogen bonds from the water present in the compound between these groups. The bands at 3,034, 2,929, and 2,922 cm^−1^ are due to the stretching of -C-H. The absorption band at 2,112 cm^−1^, present only in CQDs ^b^, can be referred to as the triple bond stretching in groups -C≡C, and -C≡N, and may also be of isothiocyanate groups. The bands at 1,654 and 1,624 cm^−1^ can be attributed to the carbonyl stretch, probably from amides present in the samples. The bands observed in the CQDs at 1,570 cm^−1^ and 1,401 cm^−1^ are attributed to asymmetric and symmetrical stretches, respectively, of the carboxylate anion (-COO^-^). The absorbed bands at 1,522 and 1,451 cm^−1^ can be attributed to -C=C stretching and -CC alkane group stretching. Amine groups and C-O bonds occasionally show vibrational energy at 1,295 cm^−1^. Bands at 1,073 and 1,031 cm^−1^ were assigned to -C-O and/or C-S group stretching. The FTIR results show that only CQDs^b^ showed stretching at 3,389 cm^−1^ and 2,112 cm^−1^ relative to the CN groups in the samples. Another distinction was the difference in relative intensities between the bands observed at 1,654 cm^−1^ and 1,451 cm^−1^, which indicated a more significant amount of carbon core containing amide groups in the CQDs^a^. The FTIR spectrum of the sludge compared to that of the CQDs makes it clear that the CQD synthesis processes converted a significant amount of organic matter in the sludge (Himaja et al., 2014; Pires et al., 2015; Meiling et al., 2016b).

### Thermal analysis

The thermoanalytical study of the sewage sludge and CQDs^a^ and CQDs^b^ obtained for the optimal synthesis methods could indicate the amount of organic matter present in the sludge, moisture content, and the presence of inorganic material, as well as evaluate the thermal behavior of the CQDs (Supplementary Table S1).

**FIGURE 5:**
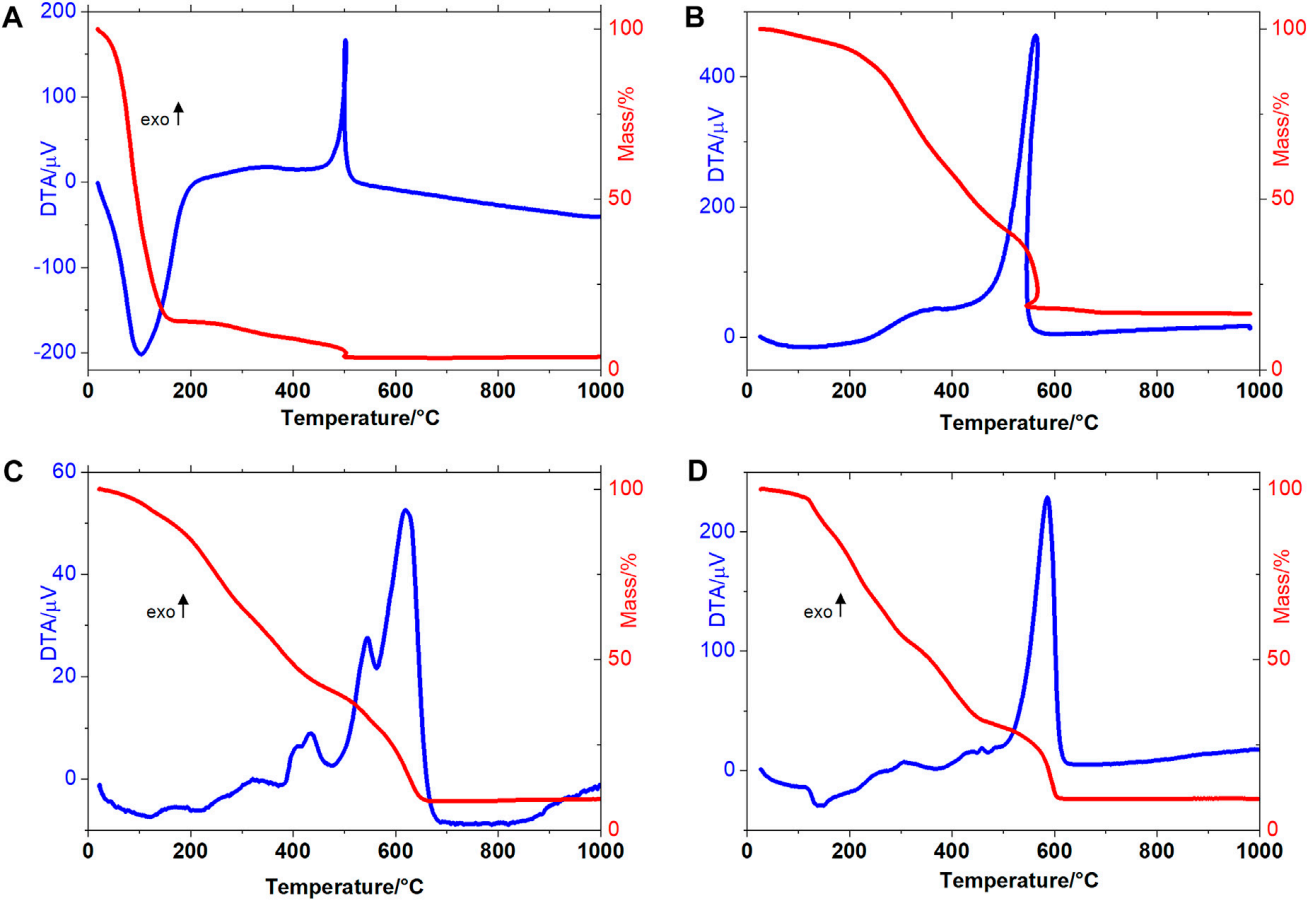
TG-DTA curves of **(A)** wet sewage sludge **(B)** dry sewage sludge **(C)** CQDs obtained *via* microwave treatment and **(D)** CQDs obtained *via* hydrothermal autoclave treatment.

The thermal decomposition of wet sewage sludge occurs in three consecutive steps, as shown in Figure 5A. The first mass loss occurs up to 150°C, with a mass loss equal to 85.54% (m/m), which refers to sludge water loss. In the second thermal decomposition step, the 8.10% mass loss is attributed to the thermal degradation of macromolecules (depolymerization and decarboxylation of proteins, lipids, carbohydrates) and other short-chain organic molecules present in sewage sludge (Ensafi et al., 2017c). In the third step of thermal decomposition (495–505°C), there is a loss of 2.20% of the material. The TG-DTA curve profile allows us to suggest a sample combustion process. The combustion phenomenon is a reliable indicator of the thermal degradation of a considerable organic charge in sewage sludge. As the masses lost in the second and third thermal decomposition steps are attributed to the thermal decomposition organic matter, it may be suggested that the evaluated sludge contains 10.30% of organic matter. The final residue had a mass equivalent to 4.16% of the initial mass, suggesting that this percentage is an inorganic component.

The TG-DTA curves of dry sewage sludge are shown in Figure 5B. The TG curve shows that the sample had a remaining water content equal to 6.01%. Due to the water leaving slowly, it was possible to observe an endotherm between 25 and 200°C. The second and third mass loss steps were equal to 51.76% and 25.46%, respectively, indicating the proportion of organic matter in the dry sewage sludge. The thermal event was observed between 500 and 600°C with exothermic peaks at 502°C (wet sludge) and 563°C (dry sludge) due to combustion of the organic matter (Campos et al., 2014). The thermogravimetry curve profile indicates that the organic matter degradation that occurred at the second step is a suitable temperature range to produce at CQDs (Monje et al., 2021).

The CQDs^a^ and CQDs^b^ TG-DTA curves can be seen in Figures 5C,D. Thus, the thermal decomposition of this material occurred in three (CQDs^a^) and four (CQDs^b^) consecutive and overlapping steps.

Thermal dehydration of the CQDs^a^ occurred in the first step between 25–137°C (Δm = 7.42%), even after the material had been subjected to the lyophilization process. The second and third mass loss steps occurred at 137–457°C (Δm = 50.37%), corresponding to the release of organic matter, probably linked to the carbon core surface. This evaluation is also indicated by the DTA curve profile obtained in these processes, corresponding to exothermic peaks at 319, 404, and 434°C. In the last thermal decomposition step, more major heat release was observed than in the previous steps, with exothermic peaks at 544°C and 620°C, so it is suggested that this step is related to carbon core burn (Δm = 33.67%). At 666°C, 8.54% stable residue was formed until 1,000°C, corresponding to the inorganic material present in the sample.

In the first step of the thermal decomposition of CQDs^b^, 2.45% of residual water was released. In the second step of thermal decomposition, an endotherm was observed at 140°C, referring to water. The third phase of thermal decomposition of CQDs^b^ occurred between 318°C and 453°C (Δm = 22.21%). An exothermic peak at 423°C was observed in the DTA curve, attributed to the release of organic matter present on the surface of the carbon core. The low energy release observed was due to the slow decomposition of the material, breakage of organic matter bonds with the cores, and simultaneous oxidative degradation. The carbonaceous core of CQDs was oxidatively degraded in the fourth step of thermal decomposition, between 453°C and 611°C, and showed intense exothermic peaks in the DTA curve at 481 and 587°C. Since the mass lost in this step was 23.28%, it may be suggested that the most significant mass of CQDs^b^ was related to the organic material on the surface of the core.

The evaluation of gases released during the thermal decomposition of sludge and CQDs^a^ and CQDs^b^ was performed using another thermoanalytical system (TG-DSC coupled FTIR). All materials showed similarities in the TG-DSC curve profile (Supplementary Figure S5) compared with the TG-DTA curves.

The thermal decomposition product observed by the evolved gas analysis (EGA) technique allowed us to indicate the molecular compounds present in the sewage sludge and on the surface of the CQDs likely responsible for the photoluminescence of each material.

**FIGURE 6:**
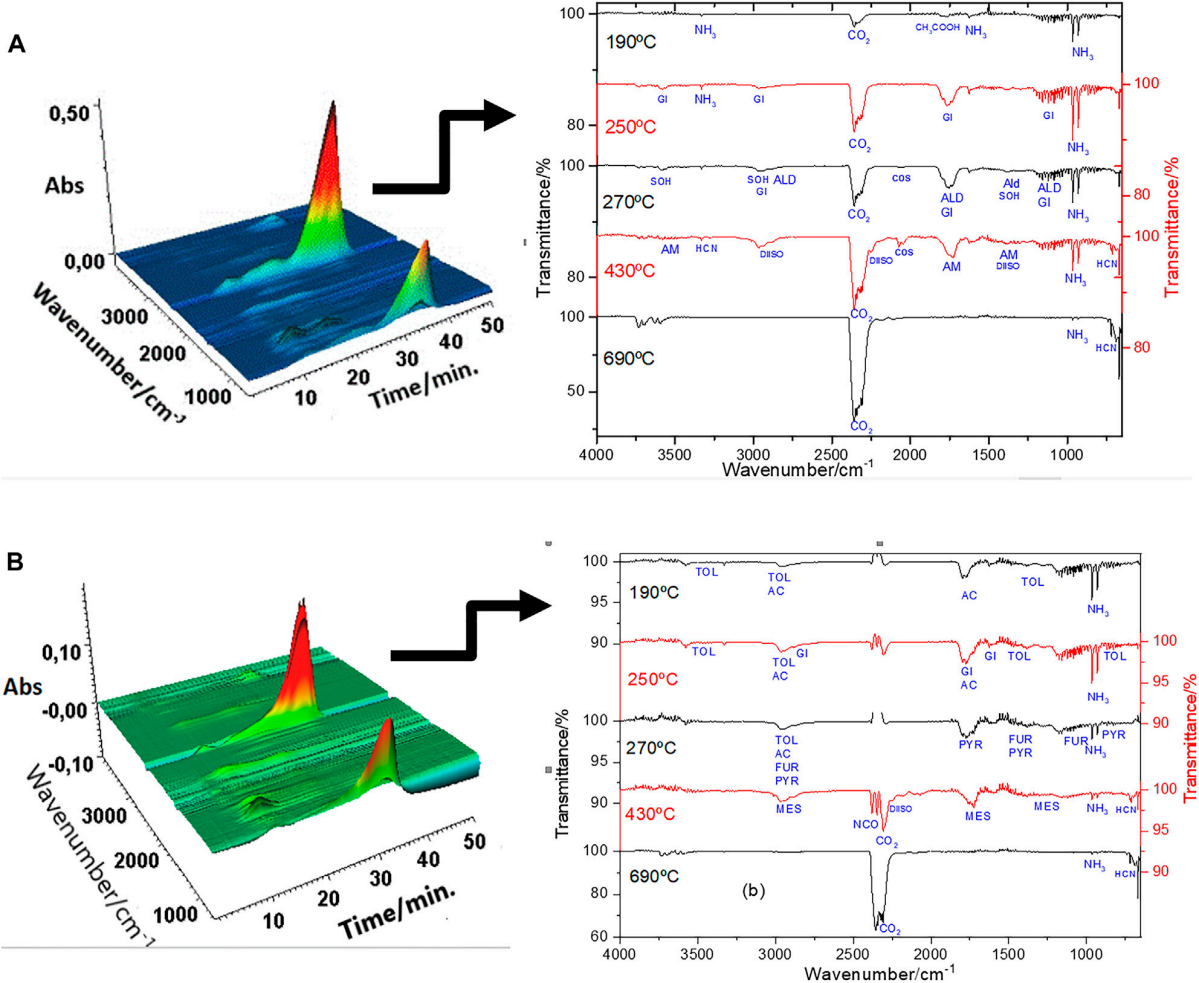
FTIR spectra from the gaseous products evolved during the thermal decomposition of **(A)** CQD^a^ and **(B)** CQD^b^.

The analysis of the sewage sludge showed the release of a large amount of carbon dioxide and ammonia, revealing the thermal decomposition of this material in the air atmosphere of the organic compounds present in the sludge, according to the literature reports (Xu et al., 2011). The evolved gas results of the CQDs synthesized by the two routes are presented in Table 1 and Figure 6. The release of an enormous variety of organic compounds in the CQDs compared to sludge is probably due to the presence of these compounds on the surface of the CQDs.

**TABLE 1:** Evolved gases observed during the thermal decomposition of CQDs^a^ and CQDs^b^.

FTIR temperature (°C)	Compounds released in CQDs^a^	Compounds released in CQDs^b^
190°C	Ammonia	N, N′-bis (1,4-dimethylpentyl) -p-phenylenediamine (TOL)
	Acetic acid (AC)	Acetic acid (AC)
250°C	Carbon dioxide	Ammonia
	Gitoxigenin (GI)	Acetic acid
	Ammonia	Gitoxigenin (GI)
		N, N-bis (1,4-dimethylphenyl) -P-phenylenidianamine (TOL)
270°C	Acetaldehyde (ALD)	Ammonia
	Gitoxigenin (GI)	Acetic acid (Ac)
	2-Mercaptoethanol (SOH)	Tetrahydrofuran (FUR)
	Carbonyl sulfide (COS)	3,4-Dimethyl-5-pyrazolone (PYR)
	Ammonia	
430°C	Carbon dioxide	Hydrogen cyanide
	Ammonia	Carbon dioxide
	Acetamide (AM)	Acetaldehyde
	Carbonyl sulfide (COS)	Ethyl M-aminobenzoate, methanesulfonic acid salt (MES)
	Isophorone diisocyanate (DIISO)	Methyl isocyanate (NCO)
	Hydrogen cyanide (HCN)	Isophorone diisocyanate (DIISO)
690°C	Hydrogen cyanide (HCN)	Carbon dioxide
	Ammonia	T-Butyl isocyanate
	Carbon monoxide	Hydrogen cyanide (HCN)
	Carbon dioxide	Carbon monoxide

**FIGURE 7:**
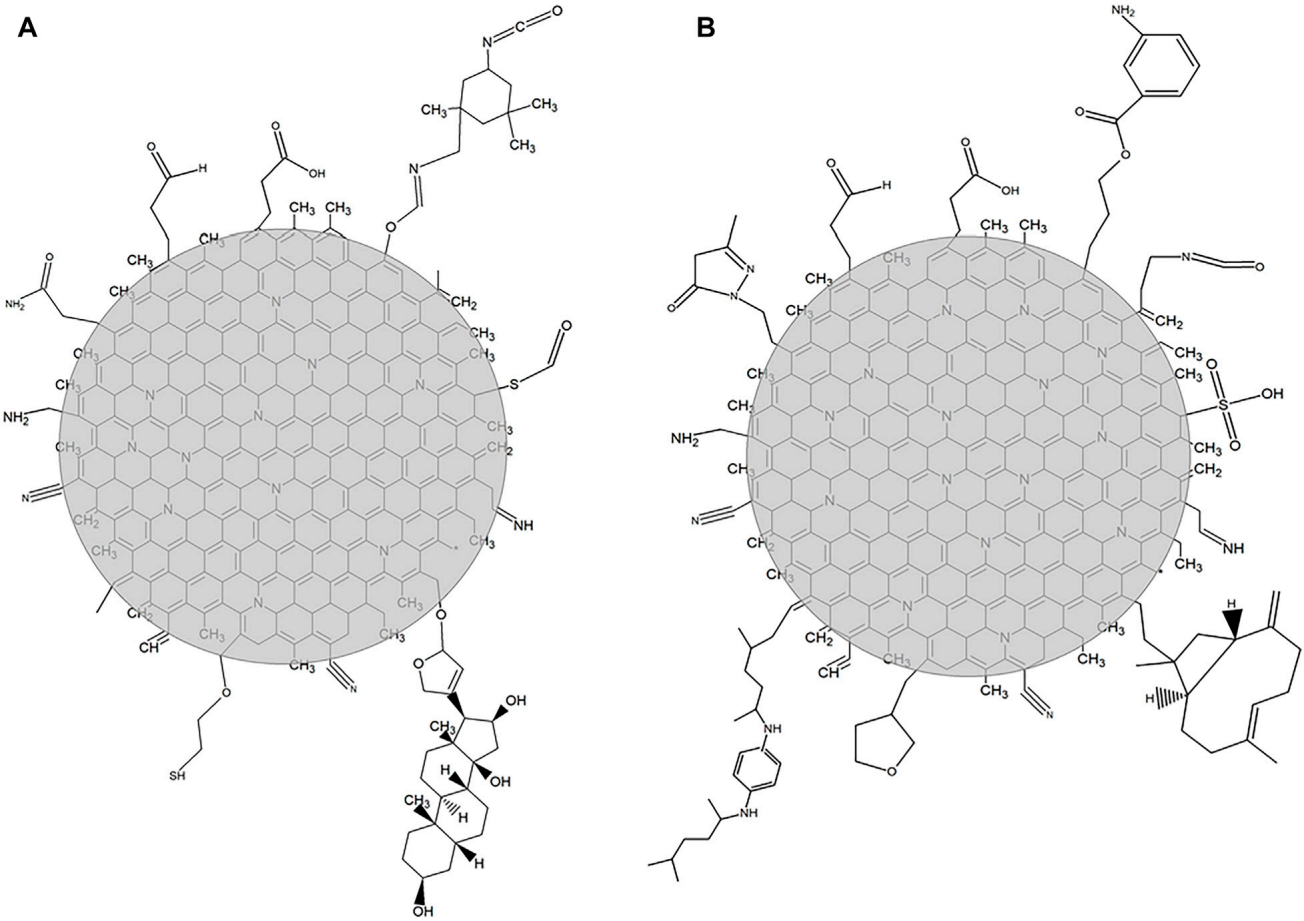
Two-dimensional representation of the carbon nucleus and surface of **(A)** CQD^a^ and **(B)** CQD^b^.

The functional group contents of elements C, S, N, O, and H verified that the CQDs were synthesized by the two routes. The organic compounds released until 430°C refer to the detachment of the material on the surface of the CQDs. Some compounds can be explained by the breakdown of complex compounds ordinarily present in sludge. 1) Acetic acid, CO_2_ and NH_3_ can be the products of amines contained in amino acids, proteins, fatty acids, and other organic compounds. The functional groups of these organic compounds were observed in the FTIR spectra (Figure 7). 2) Gitoxigenin (C_23_H_34_O_5_), ethyl M-aminobenzoate (C_9_H_11_NO_2_), and caryophyllene oxide (C_15_H_24_O) may have been obtained from complex compounds as fulvic and humic acid fragments. 3) Acetamide, acetaldehyde, 2-mercaptoethanol, methanesulfonic acid salt, carbonyl sulfide, and isophorone diisocyanate (C_12_H_18_N_2_O_2_) reproduced during the synthesis method of CQDs. 4) Tetrahydrofuran (C_4_H_8_O) was also produced in the synthesis process, but it was probably part of the initiated and unformed carbon nuclei. 5) 3,4-dimethyl-5-pyrazolone (C_5_H_8_N_2_O) release can be explained by N atoms present in the nuclei of CQDs, released at the same temperature as tetrahydrofuran.

In the thermal decomposition range close to 690°C, it was found that there was a more intense release of compounds with lower molecular weights, such as carbon monoxide and dioxide, indicating the oxidative degradation of carbonaceous nuclei of CQDs. The organic compounds, ammonia, hydrocyanic acid, and carbon dioxide were detected with both CQD synthesis routes. However, the long-chain compounds of each material differ based on the surface organization of the CQDs. The ammonia and hydrocyanic acid released at higher temperatures indicated the presence of nitrogen atoms in the nucleus. The N atom in the nucleus can be caused by the tail observed in the region of the UV-Vis spectrum between 300 and 500 nm (Figure 2). Hydrocyanic acid was also observed in the FTIR spectra of CQDs^b^ (Figure 6), indicating free hydrocyanic acid. Therefore, the conventional autoclave method is a synthesis method hazardous to produce CQDs, and its product is not indicated for use. Figure 7 shows the structural organization of the CQDs synthesized by both routes, based on the results of the EGA.

In general, the thermal analysis indicated distinct behavior between microwave and autoclave CQDs. This difference suggests a variation in the number of carbon cores formed by microwaves (33.67%) and autoclaving (23.28%), in addition to suggesting different compositions of the carbon core surfaces.

### TEM images

**FIGURE 8:**
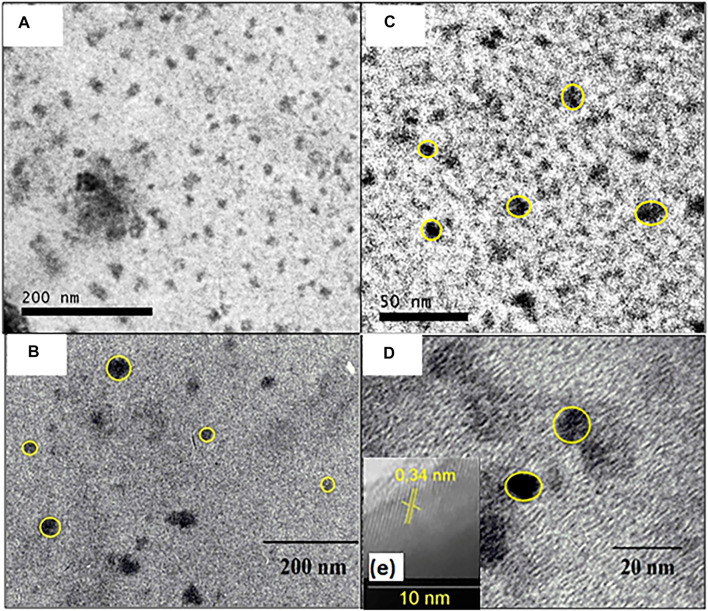
TEM images of isolated particles from CQDs prepared by heat exposure at 210°C for 30 min in enlarged microwave **(A)** 200 nm and **(C)** 20 nm; and TEM images of isolated particles from CQDs prepared by heat exposure at 210°C for 10 h in autoclave **(B)** 200 nm **(D)** 20 nm and **(E)** interplanar spacing of clustered CQDs^b^.

The CQDs^a^ images show that the isolated carbonaceous nuclei have a spherical shape and diameters of 9.69 ± 2.64 nm, as shown in Figure 8.

It can be observed that the CQDs TEM images obtained with a heat exposure time of 10 h at 210°C in the autoclave showed that they had spherical shapes and a size of 26.04 ± 6.98 nm (Figures 8A); it is also possible to observe isolated points of 10.92 ± 2.69 nm (Figure 8B). Therefore, it can be said that the different radiation sources used in the synthesis had little effect on the nanoparticle diameter. Furthermore, the extended TEM images of the clustered CQDs^b^ have a lamellar spacing of 0.34 nm, which is equal to the graphite carbon plane (002), indicating an organization of the material and suggesting that the material is crystalline, as shown in Figure 8E. Other authors have also reported interlamellar spacing values in CQDs obtained with other precursor materials (Hsu and Chang, 2012; Hu and Yang, 2014; Liu et al., 2014).

### Cellular experiment

**FIGURE 9:**
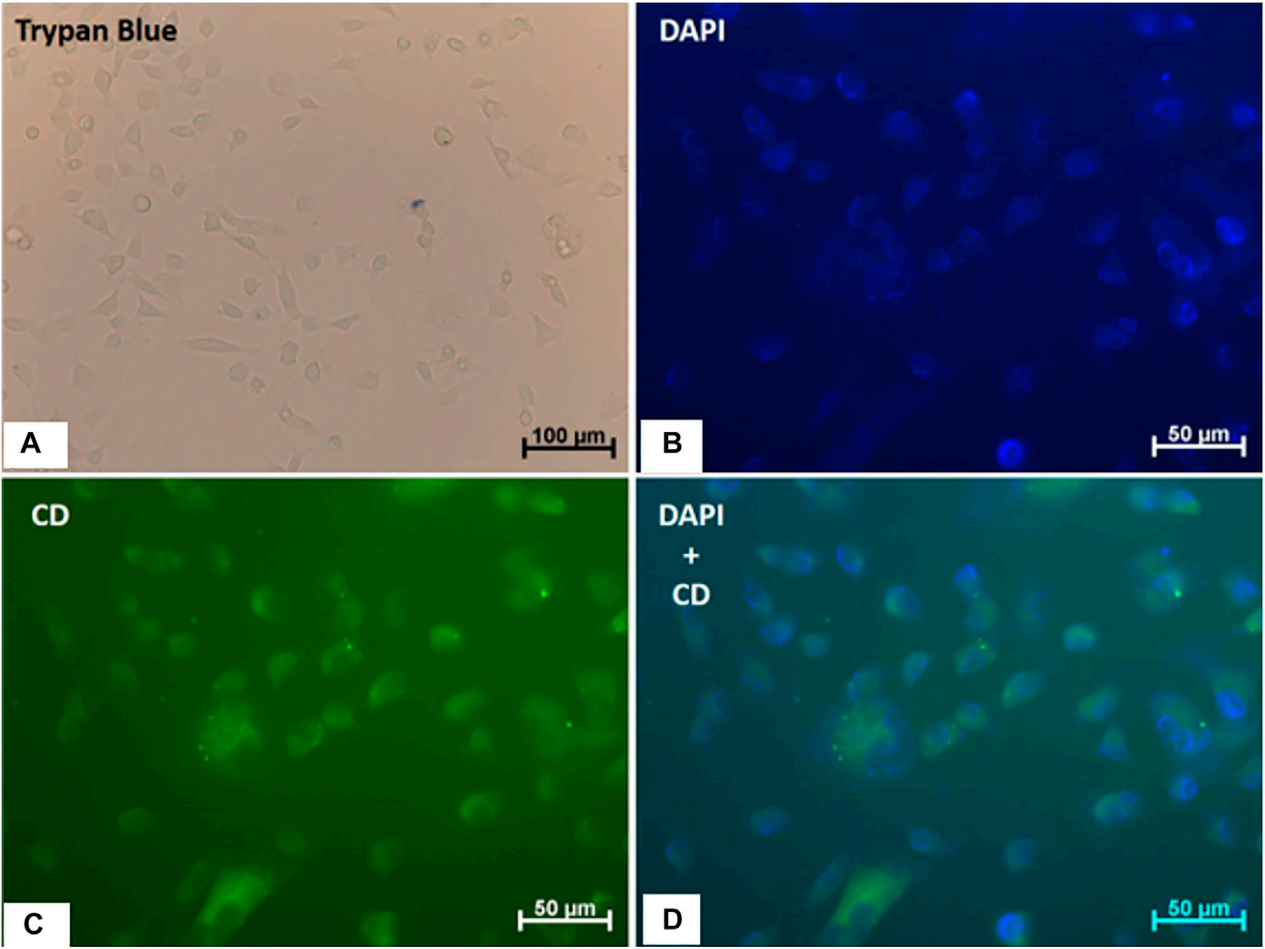
Viability, morphology and internalization of CQD^a^ by human endothelial cells (EA.hy926). **(A)** Trypan blue staining; the scale bar is 100 µm. **(B)** DAPI staining. **(C)** Fluorescence of cells treated with CQDs (0.4 mg/ml) for 1 h. **(D)** Merged images (DAPI + CQD). The scale bar is 50 µm.

*In vitro* cellular experiments were performed to study the potential of CQDs^a^ as cellular markers. The CQDs^a^ were not toxic at the evaluated concentrations, and they did not promote changes in cell morphology after uptake by EAhy 926 cells. In addition, the observed intracellular fluorescence makes them promising for use in fluorescence microscopy (Figure 9).

## Conclusion

Due to the complexity and heterogeneity of the precursor sludge, where the proportion of components or even the presence of different components may vary throughout the year (or even throughout the day), the factorial design was essential for the development of the work by combining the variables and to show the significant effects on CQD synthesis processes.

The use of thermal analysis was a differential and unpublished approach in this work to characterize CQDs obtained by microwave and autoclave treatment. Although it was possible to quantify the variation in the number of carbon cores formed by microwaves (33.67%) and autoclaving (23.28%), different compositions on the surfaces of the carbon cores were also determined. Regarding the physicochemical characterization of CQDs, different results were found for CQD^a^ and CQD^b^. However, TEM images indicated particles with diameters on the same order of magnitude, so it can be said that the synthesis methodologies affected only the components on the surface of the CQDs.

Therefore, CQDs from domestic sewage sludge are possible *via* both microwave and hydrothermal autoclave treatment synthesis routes. Nevertheless, microwave treatment is faster and simple, furthering the principles of green chemistry. The purification of these materials, or even the selectivity in the emission wavelength for their application, will occur according to the applications of these new materials.

## Data Availability

The original contributions presented in the study are included in the article/Supplementary Material, further inquiries can be directed to the corresponding authors.
