# Supplementary material for: Carbon quantum dots: An environmentally friendly and valued approach to sludge disposal
Source: Front Chem. 2022 Aug 11;10:858323. doi: 10.3389/fchem.2022.858323 (PMC9403084; doi:10.3389/fchem.2022.858323)
Supplement: Supplementary file 1 [file DataSheet1.DOCX]

CARBON QUANTUM DOTS: An environmentally friendly and valued approach to sludge disposal

Bruno L. Rossi^1^, Claúdia M. B. Andrade^1^, Eralci M. Therézio^2^, Romildo J. Ramos^2^, Leonardo G. Vasconcelos^1^, Ailton J. Terezo^1^ and Adriano B. de Siqueira^1^

^1^Genmat/Rede MT-NanoAgro- Departamento de Química/ICET, Universidade Federal de Mato Grosso, Cuiabá-MT, Brazil

^2^ Instituto de Física, Universidade Federal de Mato Grosso, Cuiabá-MT, Brazil

***Supplementary Material***

**
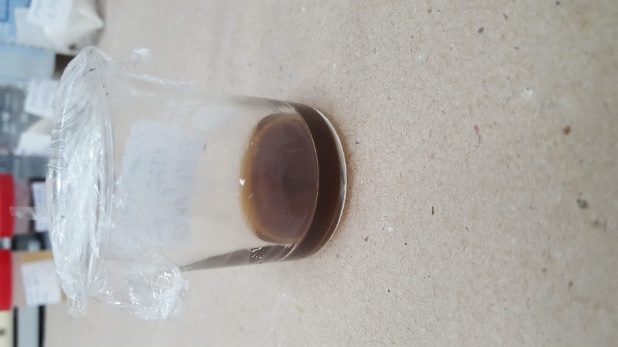
**

Figure **S1.** Photograph of CQDs solution using dry sludge as a precursor.

Figure S2 QY variation in function of synthesis time in autoclave keeping the temperature fixed at 210 °C and the initial sludge concentration of 6.25 g L^-1^.

Figure S3 The graph shows the relationship between the variation of the quantum yield with the time of exposure to microwave radiation keeping the temperature fixed at 210 °C and the initial concentration of wet sludge of 6.25 g L^-1^.

**
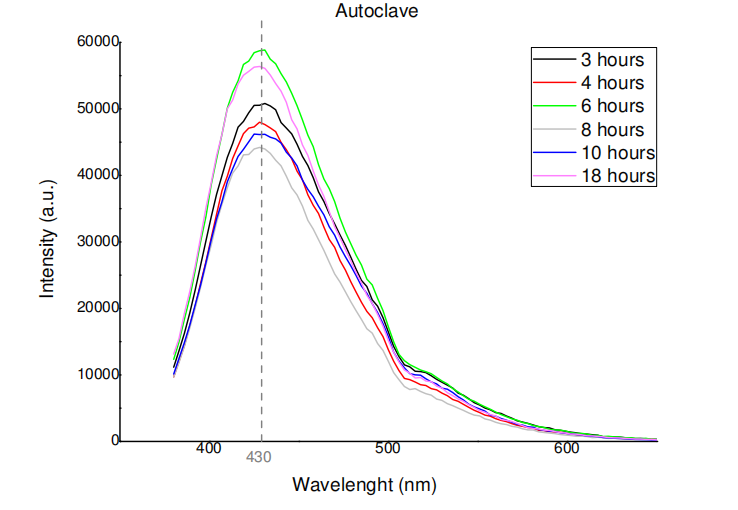

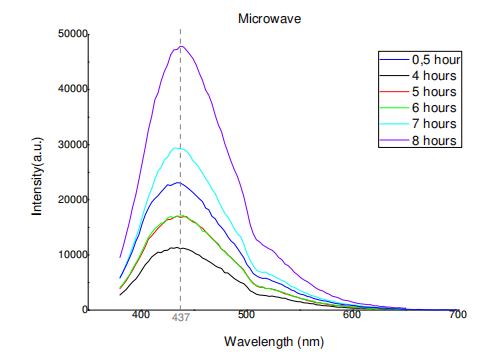
**

Figure S4. Photoluminescence spectrum of CQDs obtained by (a) autoclave (b) microwave and with different synthesis time values, with initial sludge concentration of 6.25 g L^-1^.


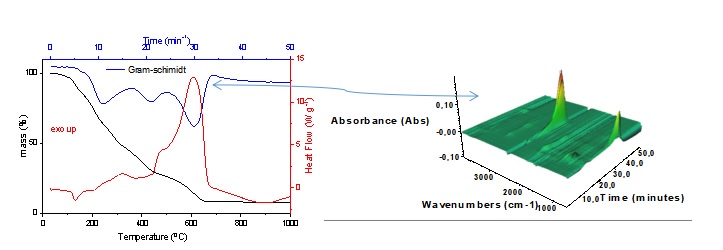


(b)


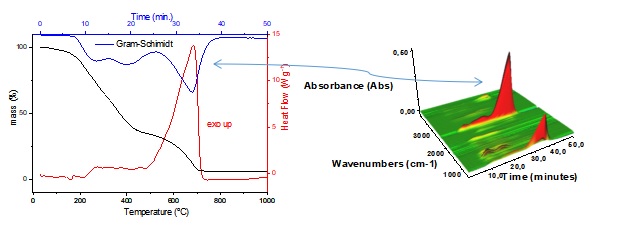


(a)

Figure S5- TG-DSC/Gram-Schimidt Curves associated with 3D spectra of (a) CQDs^a^ and (b) CQDs^b^.

Table S1. Thermoanalytical data of sewage sludge (dry and wet), and CQDs^a^ and CQDs^b^.

| **Sample** |  | **Step** | | | |
| --- | --- | --- | --- | --- | --- |
|  |  | **1ª** | **2ª** | **3ª** | **4ª** |
| Wet sludge | Θ/ºC  ∆m/%  Peak/°C | 25 – 200  85,54  104 ↓ | 200 – 495  8,10  - | 495 – 505  2,2  502↑ | - |
| Dry sludge | Θ/ºC  ∆m/%  Peak/°C | 25 – 200  6,01  - | 200 – 495  51,76  330↑ | 495 – 570  25,46  563↑ |  |
| CQDs^a^ | Θ/ºC  ∆m/%  Peak/°C | 25 – 137  7,42  126↓ | 137 – 457  50,37  319↑, 404↑, 434↑ | 457 – 666  33,67  544↑, 620↑ | _ |
| CQDs^b^ | Θ/ºC  ∆m/%  Peak/°C | 25 – 114  2,45  - | 114 – 318  42,51  140 ↓, 251↑, 309↑ | 318 – 453  22,21  423↑ | 453 – 611  23,28  459↑, 481↑,587↑ |
